# From Cell Lines to Avatars: Charting the Future of Preclinical Modeling in T-Cell Malignancies

**DOI:** 10.3390/cells15040368

**Published:** 2026-02-19

**Authors:** Pier Paolo Piccaluga, Luigi Cimmino, Valeriia Tsekhovska, Pietro Cimatti, Claudia Innocenti, Sabrina Seidenari, Giulia Calafato, Floriana J. Di Paola, Giovanni Tallini

**Affiliations:** 1 Biobank of Research, IRCCS Azienda Ospedaliero-Universitaria di Bologna, Via Massarenti, 9, 40138 Bologna, Italy; luigi.cimmino@studio.unibo.it (L.C.); florianajessica.dipaola@aosp.bo.it (F.J.D.P.); 2 Department of Medical and Surgical Sciences, Bologna University School of Medicine, 40138 Bologna, Italy; 3 Reconstructive Orthopaedic Surgery Innovative Techniques–Musculoskeletal Tissue Bank, IRCCS Istituto Ortopedico Rizzoli, 40136 Bologna, Italy; 4 Solid Tumor Molecular Pathology Laboratory, IRCCS Azienda Ospedaliero-Universitaria di Bologna, 40138 Bologna, Italy

**Keywords:** T-cell lymphoma, preclinical models, cell lines, patient-derived xenograft (PDX), translational research, peripheral T-cell lymphoma (PTCL)

## Abstract

**Highlights:**

**What are the main findings?**

- This review maps the current preclinical landscape of T-cell malignancies, showing a marked excess of T-ALL, CTCL, and ALCL cell lines contrasted with a near-complete lack of reliable models for most peripheral T-cell lymphoma (PTCL) entities.
- It delineates how newer platforms—such as patient-derived xenografts, 3D cultures, and “avatar” models—better recapitulate tumor biology and microenvironmental dependence than conventional 2D cell lines.

**What is the implication of the main finding?**

- The imbalance in available models helps explain why research and drug development are concentrated in a few T-cell lymphoma subtypes, underscoring the need for coordinated efforts to establish, authenticate, and share models of rare PTCLs.
- Integrating advanced, patient-tailored models into preclinical pipelines may improve target discovery, refine drug sensitivity testing, and ultimately support more effective precision medicine strategies in T-cell malignancies.

**Abstract:**

T-cell malignancies represent a complex spectrum of clinically and biologically heterogeneous diseases. Effective translational research and drug development are critically dependent on preclinical models that faithfully recapitulate this diversity. This review analyzes the current preclinical landscape, identifying a profound disparity between the clinical spectrum of T-cell neoplasms and the available in vitro tools. We demonstrate that the existing armamentarium of cell lines is heavily skewed, with an abundance of models for T-cell lymphoblastic leukemia/lymphoma (T-ALL), cutaneous T-cell lymphoma (CTCL), and anaplastic large cell lymphoma (ALCL). This skew is a direct result of a biological selection bias, as these entities are often driven by potent, TME-independent oncogenes (e.g., *NOTCH1* mutations, *NPM1-ALK* fusions) conducive to immortalization. Conversely, the majority of peripheral T-cell lymphoma (PTCL) subtypes, which are frequently TME-dependent and clinically aggressive, remain “preclinical orphans” with few or no authenticated models. This “preclinical void” constitutes a major bottleneck, impeding mechanistic studies and therapeutic progress. We discuss the limitations of 2D cultures and highlight the necessity of adopting advanced platforms, such as patient-derived xenografts (PDX) and 3D organoid systems. These “avatar” models preserve vital tumor heterogeneity and microenvironmental context, offering superior predictive value. The systematic development and integration of these next-generation models are essential to bridge the translational gap and advance precision medicine for all patients with T-cell malignancies.

## 1. Introduction

Human leukemia-lymphoma cell lines represent a cornerstone of modern cancer research. First established in the 1960s, these immortalized cell populations provide an unlimited, genetically stable, and globally accessible resource for investigating the fundamental pathophysiology of hematopoietic tumors [1,2,3]. Their utility in dissecting molecular pathways, identifying novel therapeutic targets, and conducting high-throughput drug screening is undisputed. However, the value of this armamentarium is contingent upon its ability to faithfully represent the vast clinical and biological diversity of the diseases under study. For T-cell malignancies, such an endeavor presents a formidable challenge [4].

T-cell neoplasms are not a monolithic entity but rather a complex collection of dozens of distinct diseases, as defined by the World Health Organization (WHO) and International Consensus Classification (ICC) systems [5]. These malignancies arise from T-lymphocytes at various stages of differentiation, ranging from immature thymic precursors, which give rise to T-cell lymphoblastic leukemia/lymphoma (T-ALL/LBL), to a wide spectrum of mature, post-thymic T-cells that form the basis of peripheral T-cell lymphomas (PTCLs) [6,7,8]. Our group and others have dedicated significant effort to elucidating the profound heterogeneity within the PTCL category, which is characterized by immense morphological, immunophenotypic, and molecular diversity [9]. This complexity makes PTCLs exceptionally difficult to classify, diagnose, and treat, earning them the unfortunate designation of “orphan diseases” [10,11].

**Figure 1:**
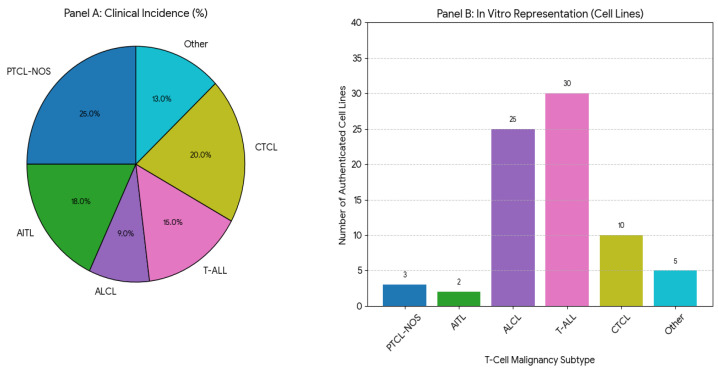
Disparity in Preclinical Models: Clinical Incidence vs. In Vitro Representation of T-Cell Malignancies. Panel A illustrates the approximate clinical incidence of major T-cell malignancy subtypes. Panel B shows the number of currently available, authenticated cell line models for these subtypes. The marked imbalance highlights a major research gap, particularly for Peripheral T-cell lymphoma, not otherwise specified (PTCL-NOS), Angioimmunoblastic T-cell lymphoma (AITL), and Hepatosplenic T-cell lymphoma (HSTCL), which lack suitable in vitro models for mechanistic and preclinical studies. For cutaneous T-cell lymphomas (CTCL), please note that they represent a heterogeneous category, accounting for different entities, only a few of which are represented by cell lines.

This review sought to show that a critical disparity exists between the clinical diversity of T-cell malignancies and the preclinical models available to study them. The landscape of available cell lines is heavily skewed, with an abundance of well-characterized models for a few specific subtypes—namely, T-ALL/LBL, cutaneous T-cell lymphoma (CTCL), and anaplastic large cell lymphoma (ALCL). In stark contrast, for the majority of PTCL entities, which are often highly aggressive and associated with dismal prognoses, reliable and authenticated cell line models are either exceedingly rare or entirely non-existent [11,12]. This imbalance is not a mere artifact of historical collection but a direct consequence of the underlying biology of these tumors. Malignancies such as ALK-positive ALCL and many T-ALLs are driven by potent oncogenes, like the *NPM1-ALK* fusion protein or activating *NOTCH1* mutations, which confer robust, cytokine-independent proliferative signals that are highly conducive to establishment and maintenance in artificial in vitro culture conditions [13]. Conversely, many rare PTCLs, such as angioimmunoblastic T-cell lymphoma (AITL), are intrinsically dependent on a complex tumor microenvironment (TME) for survival and proliferation—a condition that is lost in standard two-dimensional culture systems. This biological selection bias has created a vicious cycle: the availability of models drives research and drug discovery, leading to disproportionate progress in ALCL and T-ALL [14], while the entities lacking models remain neglected, perpetuating their status as preclinical and clinical orphans (Figure 1).

## 2. The Established Landscape: Available T-Cell Malignancy Cell Lines

The current collection of T-cell malignancy cell lines, curated by repositories such as the American Type Culture Collection (ATCC) and the German Collection of Microorganisms and Cell Cultures (DSMZ), reflects this skewed distribution. A systematic review reveals a clear pattern of abundance for some entities and a profound void for others (Table 1, Table 2 and Table 3).

### 2.1. Models for Immature T-Cell Neoplasms: T-Lymphoblastic Leukemia/Lymphoma (T-ALL/LBL)

T-ALL/LBL are aggressive neoplasms of T-cell precursors, accounting for approximately 15% of pediatric and 25% of adult ALL cases [7,15,16]. The molecular pathogenesis is characterized by a multi-step accumulation of genetic lesions. Key events include the aberrant expression of oncogenic transcription factors (e.g., *TAL1*, *TLX1*, *HOXA*), which often arise from chromosomal translocations involving T-cell receptor (*TCR*) loci and define distinct molecular subgroups that correspond to specific stages of T-cell developmental arrest [15,16]. These events are frequently coupled with hyperactive NOTCH1 signaling, due to activating mutations in over 60% of cases, and loss of the *CDKN2A/B* tumor suppressor locus in up to 70% of cases [15,17]. The strong proliferative signals conferred by these mutations have facilitated the establishment of numerous cell lines (Table 1).

A comprehensive analysis of 16 widely used T-ALL cell lines demonstrated that, despite their common classification, each line possesses a unique immunophenotype and TCR gene rearrangement status. This study proposed a categorization into pro-T, pre-T, cortical T, and mature T differentiation stages, underscoring the heterogeneity that these models collectively represent [18].

### 2.2. Models for Cutaneous T-Cell Lymphomas (CTCL)

CTCLs are a group of overall rare extranodal non-Hodgkin lymphomas defined by the infiltration of malignant T-cells into the skin, with Mycosis Fungoides (MF) and the related leukemic form, Sézary Syndrome (SS), being the most prevalent subtypes [19]. Several key cell lines have been established, primarily from patients with advanced-stage, aggressive disease (Table 2) [20,21]. These models have been instrumental in preclinical studies, particularly for evaluating novel therapeutic agents. For instance, the HUT 78 cell line was used to demonstrate the sensitivity of T-cell lymphoma to the histone deacetylase inhibitor (HDACi) depsipeptide [22], providing a key preclinical rationale for the development of this drug class for T-cell malignancies [23]. It should be noted, however, that CTCL remains an orphan disease and establishing reliable models is difficult, since the disease exhibits significant dependence on the tumor microenvironment (TME), such as interactions with keratinocytes and fibroblasts.

### 2.3. Models for Anaplastic Large Cell Lymphoma (ALCL)

**Figure 2:**
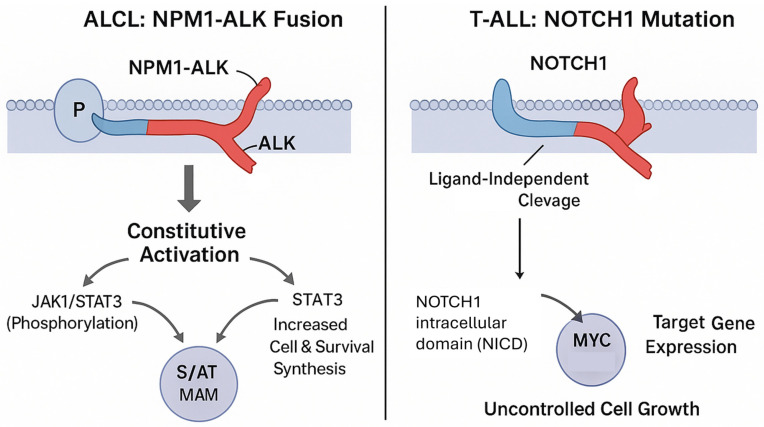
Constitutively active signaling pathways in amenable T-cell lymphoma subtypes. This diagram illustrates potent, self-sustaining oncogenic signaling mechanisms in ALCL (NPM1–ALK fusion) and T-ALL (NOTCH1 mutation) that drive robust cell line establishment and survival in vitro. In ALCL, the NPM1–ALK fusion results in constitutive activation of the JAK/STAT3 and PI3K/AKT/mTOR pathways, promoting MYC-driven proliferation and survival. In T-ALL, activating NOTCH1 mutations lead to ligand-independent cleavage and release of the NOTCH1 intracellular domain (NICD), which interacts with CSL/MAML to upregulate target genes such as MYC and CYCLIN D1, sustaining uncontrolled cell growth. This highlights a biological selection bias favoring cell line establishment from subtypes with strong autonomous signaling activity, such as ALCL and T-ALL.

ALCL is a mature T-cell lymphoma characterized by strong expression of the CD30 antigen [24]. It is divided into two main categories based on the expression of Anaplastic Lymphoma Kinase (ALK). ALK-positive ALCL, which is more common in younger patients, is defined by chromosomal rearrangements involving the *ALK* gene on chromosome 2p23, most frequently the t(2;5)(p23;q35) translocation that generates the potent *NPM1-ALK* fusion oncogene [25]. The constitutive kinase activity of NPM1-ALK drives robust downstream signaling through pathways including JAK/STAT and PI3K/AKT, promoting cell survival and proliferation [26]. This powerful oncogenic driver has made ALK+ ALCL one of the most successfully modeled PTCL subtypes, with a wealth of available cell lines (Table 3) [27]. These models have proven essential not only for in vitro studies but also for confirming tumorigenicity in vivo, as demonstrated by the characterization of novel lines in SCID mice [28]. In contrast, ALK-negative ALCL, which lacks *ALK* rearrangements and generally has a poorer prognosis, is less well-represented by cell line models, though some exist (Table 3; Figure 2) [5,29,30].

### 2.4. The Void: Scarcity of Models for Other Peripheral T-Cell Lymphomas (PTCL)

Beyond ALCL and CTCL, the landscape of preclinical models for mature T-cell malignancies is remarkably sparse. This gap is particularly acute for the diverse and aggressive subtypes that fall under the PTCL umbrella. As our group has demonstrated through extensive gene expression profiling studies, many of these entities arise from distinct functional T-cell subsets, such as the derivation of AITL from T-follicular helper (TFH) cells [9,31]. This cellular origin implies a profound dependence on signals from a complex TME, a feature that is lost in conventional cell culture and likely explains the extreme difficulty in establishing stable cell lines for these diseases.

It should be noted that the precise number of authenticated cell lines may vary across different literature sources and repository updates. However, in general, it is possible to claim that there are no widely available, authenticated cell lines for many of the most challenging PTCLs, including, among others, angioimmunoblastic T-cell lymphoma (AITL), hepatosplenic T-cell lymphoma (HSTCL) [32], enteropathy-associated T-cell lymphoma (EATL) and extranodal NK/T-cell lymphoma, nasal type (ENKTL) [33].

A notable and recent exception is the T8ML-1 cell line, the first authenticated model for PTCL, not otherwise specified (PTCL-NOS), harboring the recurrent t(14;19)(q11.2;q13.3) translocation [30,34]. This translocation results in the juxtaposition of the *TCR alpha* (*TRA@*) locus and the *PVRL2* gene, leading to its overexpression [34]. The successful characterization of T8ML-1 provides a crucial and unique tool for studying this specific genetic subtype of PTCL-NOS, but it also highlights the profound scarcity of models for this “wastebasket” category of PTCL [34]. The complexity of this category is further exemplified by cell lines like FE-PD, a CD30-positive line originally established from a patient diagnosed with Hodgkin’s disease, later reclassified as an aggressive anaplastic large cell lymphoma, and now considered a model for ALK-negative ALCL or CD30+ PTCL-NOS [35,36].

This striking imbalance between diseases and models has tangible consequences. The entities with robust in vitro models, such as ALCL and CTCL, are precisely those that have seen some notable progress in targeted therapy, including the clinical approval of ALK inhibitors and the early indication of HDAC inhibitors, respectively [13]. In contrast, diseases like PTCL/NOS, AITL and HSTCL, for which cell lines are so scant, continue to be treated with conventional, non-specific chemotherapy regimens with poor outcomes [8,11,12,37,38,39]. This correlation underscores a direct link: the availability of a preclinical model system is a critical prerequisite for successful translational research and the development of targeted therapies.

**Table 1:** Characteristics of selected T-lymphoblastic leukemia/lymphoma (T-ALL/LBL) cell lines.

Cell Line	ATCC/DSMZ No.	Patient Origin	Key Genetic Alterations	Immunophenotypic Stage	Key Reference(s) (PMID)
Jurkat	TIB-152/ACC 282	14Y, M, Relapsed T-ALL	PTEN null, CDKN2A del, TP53 R196*	Cortical/Mature T	[5]
CCRF-CEM	CCL-119/ACC 240	3Y, F, Relapsed T-ALL	FBXW7 R465C, TP53 R248Q	Cortical T	[5]
MOLT-3	CRL-1552/ACC 316	19Y, M, Relapsed T-ALL	FBXW7 R505C, TP53 R248Q	Cortical T	[5]
SUP-T1	CRL-1942/ACC 140	8Y, M, Relapsed T-LBL	FBXW7 R465H, TP53 null	Mature T (CD4+/CD8+)	[7]
DND-41	ACC 525	13Y, M, T-ALL	TLX3-BCL11B fusion, IL7R ins, NOTCH1 mut	Cortical T (Type III)	[18,40]
HPB-ALL	ACC 483	14Y, M, T-ALL	NOTCH1 mut, TP53 C124*, FBXW7 mut	Cortical T (CD4+/CD8+)	[18,40]

Abbreviations: Y, year; M, male; F, female; del, deletion.

**Table 2:** Characteristics of selected cutaneous T-cell lymphoma (CTCL) cell lines.

Cell Line	ATCC/DSMZ No.	Histotype/Mature T-Cell Origin	HTLV-1 Status	Key Genetic Alterations	Key Reference(s) (PMID)
HUT 78	TIB-161/ACC 398	Sézary Syndrome (Central Memory T-cell, TCM)	Negative	TP53 C176F	[8]
H9	HTB-176	CTCL (HUT 78 clone; TCM phenotype)	Negative	CDKN2A del, RB1 del, TP53 R196*	[4]
HH	CRL-2105/ACC 707	Aggressive CTCL (Non-MF/SS; Mature Cytotoxic phenotype)	Negative	FOXK2::TP63 fusion, TP53 mut	[10,31]
MJ [G11]	CRL-8294	CTCL (Mature CD4+; Skin-homing phenotype)	Positive	-	[10]
MyLa	ACC 285	Mycosis Fungoides (Skin-resident Memory, TRM)	Negative	CDKN2A del, PTEN mut	[20]

Abbreviations: del, deletion; mut, mutation.

**Table 3:** Characteristics of selected anaplastic large cell lymphoma (ALCL) and other peripheral T-cell lymphoma (PTCL) cell lines.

Cell Line	ATCC/DSMZ No.	Histotype	Key Genetic Alterations	Key Reference(s) (PMID)
SU-DHL-1	CRL-2955/ACC 356	ALCL, ALK+	NPM1-ALK t(2;5), TP53 R273H	[9]
Karpas 299	ACC 31	ALCL, ALK+	NPM1-ALK t(2;5)	[40]
SUP-M2	ACC 509	ALCL, ALK+	NPM1-ALK t(2;5)	[41]
KI-JK	ACC 695	ALCL, ALK+	NPM1-ALK t(2;5)	[25]
SR-786	ACC 369	ALCL, ALK+	NPM1-ALK t(2;5)	[2,13,28]
DEL	ACC 338	ALCL, ALK+	NPM1-ALK t(2;5)	[2,13,28,35]
FE-PD	-	PTCL-NOS/ALK-ALCL	JAK1 G1097V, STAT3 G618R	[29,35]
TLBR-2	ACC 905	BIA-ALCL, ALK-	STAT3 D661Y	[29]
T8ML-1	-	PTCL-NOS	TRA@::PVRL2 t(14;19)	[34]

Abbreviations: BIA-ALCL, Breast Implant-Associated ALCL; PTCL-NOS, Peripheral T-cell lymphoma, not otherwise specified.

## 3. Utility and Limitations: The Role of Cell Lines in Translational Research and Drug Screening

The available T-cell lymphoma cell lines, though limited in scope, have been indispensable for major advances in both basic and translational research. The ALCL cell lines, for example, were fundamental in validating ALK as a bona fide therapeutic target, elucidating its downstream signaling pathways, and serving as the primary preclinical platform for testing the first generation of ALK inhibitors like crizotinib [13,26]. Similarly, CTCL cell lines have been crucial for demonstrating the potent anti-lymphoma activity of HDACis, providing the preclinical proof-of-concept that paved the way for their successful clinical development and approval [22]. In T-ALL, cell lines have been workhorses for dissecting the complex transcriptional networks downstream of NOTCH1 activation and for evaluating the therapeutic potential of gamma-secretase inhibitors [15,40,42]. On the other hand, the lack of consistent models has so far failed to translate the genetic findings into efficient targeted approaches and valuable biomarkers. A remarkable example is how the strong evidence of PDGFRA expression in most PTCL/NOS could not translate into effective treatments with tyrosine kinase inhibitors, even if the only available model showed a striking dependence on its signaling. Similarly, VEGFR inhibition was not tested pre clinically in AITL, and still the evidence of T-cell receptor activation through mutations and translocations could not be translated into effective inhibition.

## 4. Key Scientific Questions Driving the Need for Better Models

While invaluable, the current armamentarium of cell lines primarily allows researchers to address questions pertinent to the biology of self-propagating, TME-independent tumors. Key scientific questions that have been successfully explored using these models include several issues. First, the dissection of oncogenic pathways. Cell lines like Karpas 299 and SU-DHL-1 have been instrumental in mapping the signaling cascades downstream of the NPM1-ALK fusion protein, identifying critical dependencies on pathways like JAK/STAT and PI3K/AKT [26,43]. Similarly, T-ALL lines have clarified the central role of NOTCH1 signaling in driving leukemogenesis [40,42,44]. Second, regarding target validation and preclinical drug screening, the availability of these models provides a high-throughput platform to test the efficacy of targeted drugs. The validation of ALK inhibitors in ALCL and HDAC inhibitors in CTCL are prime examples of successful translational research pipelines built upon cell line models. Third, cell lines are powerful tools for elucidating how novel drugs exert their anti-tumor effects and for investigating the molecular mechanisms that lead to acquired drug resistance.

Unfortunately, the most pressing challenges in the field of T-cell lymphomas lie in the entities for which these models are lacking. The scarcity of representative cell lines for the majority of PTCL subtypes means that numerous fundamental scientific questions remain largely unanswered, underscoring the urgent need for new and more sophisticated preclinical tools [41]. One of the most challenging issues in PTCLs is the definition of the role of the tumor microenvironment (TME) [45]. Many PTCLs, particularly AITL, are defined by a complex and pathologically integral TME. This TME consists of a dense network of non-malignant cells, including follicular dendritic cells, endothelial cells, and reactive B-cells, which provide essential survival signals through cytokine secretion and direct cell-to-cell contact. Standard cell lines, which are selected for growth in isolation, cannot be used to study the critical survival signals provided by the TME or to test therapies that target these tumor-stroma interactions. In addition, the cell of origin and even the normal counterparts of PTCLs are still largely unknown. While gene expression profiling has linked entities like AITL to T-follicular helper cells [46,47], the exact developmental stage and functional state of the cell of origin for most PTCL-NOS cases remain obscure [9,31,48,49]. Functional models derived from these specific subtypes are required to validate these hypotheses and understand the initial steps of transformation.

Another valuable point is understanding how rare, subtype-defining genetic lesions drive lymphomagenesis. The discovery of recurrent alterations like the *TRA@::PVRL2* translocation in a subset of PTCL-NOS was a major step forward, and the T8ML-1 cell line provides a unique model to study its consequences [34]. However, for dozens of other recurrent but rare mutations, no models exist, preventing any functional investigation into their pathogenic role [50].

Of note, the lack of models for entities like AITL and HSTCL [12] creates a vicious cycle: without a platform for preclinical testing, rational drug development is stalled, and these patients continue to be treated with non-specific chemotherapy that yields poor outcomes [11,37,39,50]. Similarly, the mechanisms of intrinsic and acquired chemoresistance in PTCL are not well defined. Most PTCLs are notoriously resistant to standard chemotherapy regimens. Understanding the underlying biological basis for this resistance requires models that faithfully recapitulate the genetic and phenotypic heterogeneity of patient tumors, a feature that clonally selected, long-passaged cell lines often lose. Finally, the development of treatments like CAR-T cells requires preclinical models that allow for the assessment of immune-mediated killing [51]. This is a significant challenge in systems lacking a competent immune system or the relevant tumor antigens, highlighting the need for syngeneic models or humanized PDX models [52,53].

Addressing these fundamental questions is not possible with the current, skewed collection of cell lines. It necessitates a concerted effort to develop a new generation of preclinical models, including authenticated cell lines from rare entities and, critically, PDX models that better preserve the biological complexity of the primary disease [54].

Despite these successes, the inherent limitations of cell line models are significant and well-documented. Having been selected for robust growth in artificial, two-dimensional culture, they represent a clonally homogeneous population that fails to capture the extensive intra-tumoral heterogeneity that is a hallmark of cancer in patients and a major driver of therapeutic resistance [55,56]. Furthermore, these models are completely divorced from the native TME, which provides critical survival signals and contributes to drug resistance [57]. This is a particularly glaring deficiency for lymphomas like AITL, where the TME is not merely supportive but is a defining pathological feature of the disease [58]. The cumulative result of these shortcomings is a poor predictive value for clinical success; promising results from cell line studies frequently fail to translate into patient benefit, with over 90% of drugs that show preclinical activity ultimately failing in clinical trials [51,57].

The translational research efforts of our own group, which have focused on identifying molecular signatures and prognostic markers in PTCL, are directly impacted by this lack of representative models [11]. While our work and that of others have identified promising therapeutic avenues, such as targeting aberrantly activated tyrosine kinases or epigenetic regulators, the ability to rigorously test these hypotheses in a disease-specific context is severely hampered by the absence of appropriate preclinical tools [26,59].

## 5. Future Perspectives: Expanding the Preclinical Toolkit with Patient-Derived Xenografts (PDX)

Addressing the profound deficit in preclinical models for T-cell lymphomas requires a multi-pronged approach. First, a renewed, concerted international effort to establish novel cell lines from underrepresented PTCL subtypes is warranted, perhaps employing advanced co-culture or three-dimensional organoid systems that better recapitulate the TME [41]. The rigorous authentication and characterization of the T8ML-1 cell line serve as a valuable blueprint for such endeavors [34].

**Figure 3:**
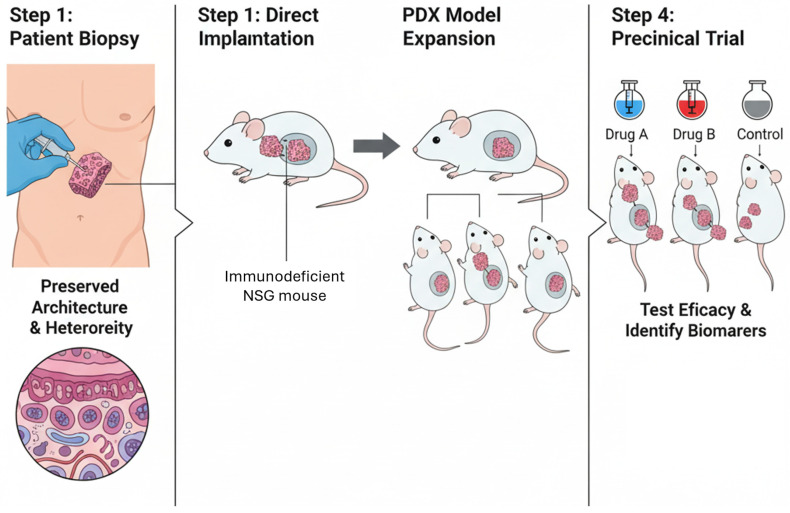
Schematic representation of Patient-Derived Xenograft (PDX) generation and application in translational research. Patient tumor biopsies are directly implanted into immunodeficient NSG mice, preserving the original tumor architecture and heterogeneity. After successful engraftment, PDX models are expanded for subsequent preclinical testing. These “mouse avatar” models allow evaluation of drug efficacy (e.g., Drug A, Drug B, Control) and identification of predictive biomarkers of response or resistance.

However, the most promising and immediately impactful strategy is the development and widespread adoption of patient-derived xenograft (PDX) models [53,60,61]. Created by the direct implantation of fresh patient tumor tissue into severely immunodeficient mice (e.g., NOD-scid IL2Rgamma null or NSG mice), PDX models circumvent many of the critical limitations of traditional cell lines [54,55,61]. Direct transplantation of fresh tissue (rather than frozen or processed cells) not only largely preserves the original tumor architecture, maintains the cellular and genetic heterogeneity of the primary disease, but also retains key components of the human TME, including stromal and vascular elements. This maximizes the viability of both the malignant clones and the associated stromal elements, but it requires immediate proximity to clinical facilities [52,55]. Consequently, PDX models have demonstrated a significantly higher predictive value for clinical drug responses, making them a superior platform for translational research (Figure 3) [52,53].

Overall, PDX models offer the advantage of preserving tumor architecture and heterogeneity; however, they are limited by high costs, long establishment times, and the eventual loss of the human TME as it is replaced by murine stroma.

The establishment of public repositories has been a transformative step, democratizing access to these powerful but resource-intensive models. The Public Repository of Xenografts (PRoXe), founded at the Dana-Farber Cancer Institute, is a landmark initiative that has generated and made available a large collection of well-characterized hematologic malignancy PDXs [60,62,63]. Crucially, this and other efforts have succeeded in creating stable, transplantable PDX models for T-cell lymphomas that have historically been impossible to culture in vitro [62].

An AITL PDX has been successfully established, maintaining the characteristic CD4+/PD1+ immunophenotype of TFH cells and harboring hallmark mutations in genes such as *TET2* [62]. The ability to propagate this TME-dependent disease in vivo is a breakthrough, finally providing a renewable model to study AITL biology and test therapies targeted against its unique genetic drivers, such as mutations in *RHOA*, *TET2*, and *IDH2* [5,39]. In addition, a PTCL-NOS PDX was shown to retain the clonal TCR gene rearrangement of the primary tumor and recapitulate a complex microenvironment containing non-malignant human T- and B-cells [62].

PDX models of both systemic and breast implant-associated ALK-negative ALCL have been developed, providing invaluable tools for a disease with a poorer prognosis than its ALK-positive counterpart [29,30].

Finally, a PDX model derived from an SS patient demonstrated appropriate disease dissemination, with malignant cells trafficking to the skin, spleen, and bone marrow, thus faithfully recapitulating the systemic nature of the disease [62].

It should be noted, however, that PDX models offer the advantage of preserving tumor architecture and heterogeneity; however, they are limited by high costs, long establishment times, and the eventual loss of the human TME as it is replaced by murine stroma. On the other hand, in addition to PDX, emerging platforms such as 3D organoids and microfluidic co-culture systems offer promising avenues to study TME interactions in a high-throughput manner.

The availability of these models enables a paradigm shift in preclinical drug development toward “co-clinical trials” or “mouse avatar” studies. In this approach, cohorts of mice bearing PDXs from different patients can be used to conduct randomized, phase II-like trials, allowing for the robust evaluation of novel agents and the identification of biomarkers that predict response or resistance [55,64].

## 6. Conclusions

The landscape of preclinical models for T-cell malignancies is one of stark contrasts. While a rich and well-characterized collection of cell lines exists for T-ALL, CTCL, and ALK-positive ALCL, this abundance is a direct reflection of a biological selection bias favoring tumors with potent, culture-permissive oncogenic drivers. For the majority of PTCL subtypes—many of which are rare, aggressive, and lack effective therapies—the preclinical toolkit is profoundly deficient. This gap has created a significant bottleneck in research, hindering our understanding of disease biology and impeding the development of novel targeted therapies. To break this cycle of neglect, the field must move beyond its reliance on a small, biased panel of historical cell lines. The future of translational research in T-cell lymphoma lies in the development and utilization of more clinically relevant models. Patient-derived xenografts, which preserve the heterogeneity and microenvironment of the original tumor, represent the most powerful tool currently available. The expansion and broad accessibility of PDX models through public repositories like PRoXe offer the most promising path forward to bridge the translational gap, enabling biologically informed drug development and ultimately improving outcomes for patients with the full spectrum of T-cell lymphomas and leukemias [60,63].

## Data Availability

Not applicable.
